# New Perspectives in the Fight Against Multidrug-Resistant Bacteria: The Potential of Endolysin Biocomposites

**DOI:** 10.3390/antibiotics14050457

**Published:** 2025-04-30

**Authors:** Carlos E. Camacho-González, Cesar S. Cardona-Felix, Alejandro Pérez-Larios, Víctor M. Zamora-Gasga, Sonia G. Sáyago-Ayerdi, Jorge A. Sánchez-Burgos

**Affiliations:** 1 Food Research Laboratory, Technological Institute of Tepic, National Technological Institute of Mexico, Instituto Tecnológico Avenue No. 2595, Lagos del Country, Tepic C.P. 63175, Nayarit, Mexico; charly_cecg@hotmail.com (C.E.C.-G.); vzamora@ittepic.edu.mx (V.M.Z.-G.); ssayago@ittepic.edu.mx (S.G.S.-A.); 2 Instituto Politécnico Nacional, CICIMAR, Av. Instituto Politécnico Nacional S/N, Col. Playa Palo de Santa Rita, La Paz C.P. 23096, Baja California, Mexico; ccardona@ipn.mx; 3 Centro Universitario de Los Altos, Universidad de Guadalajara, Av. Rafael Casillas Aceves No. 1200, Tepatitlán de Morelos C.P. 47620, Jalisco, Mexico; alarios@cualtos.udg.mx

**Keywords:** endolysins, colonic fermentation, alginate oligosaccharides, modified cellulose

## Abstract

The growing threat of multidrug-resistant bacteria requires innovative therapies beyond traditional antibiotics. This review highlights the potential of endolysin biocomposites using alginate oligosaccharides (AOSs) and modified cellulose (CL) as stabilizers. AOSs could enhance endolysin stability and potentially support colonic fermentation, producing short-chain fatty acids that may synergize with endolysins to combat pathogens and improve gut health. KZ144 and LysPA26 are proposed as optimal candidates for their broad pH range, divalent cation tolerance, and potential effectiveness against Gram-positive and Gram-negative pathogens. Integrating AOSs and CL into biocomposites could offer a novel dual-action strategy against gastrointestinal diseases while potentially reducing antibiotic dependence.

## 1. Introduction

Antibiotic resistance has emerged as a critical challenge to human health. Projections estimate that by 2050 [1], approximately 10 million deaths could be attributed to this issue, largely associated with prolonged hospitalizations [1]. This situation is further exacerbated by the excessive and indiscriminate use of antibiotics across various sectors. Consequently, the rapid dissemination of antibiotic resistance affects not only developing nations but also industrialized countries, resulting in significant economic burdens on public health systems [2].

Since the progress of new antibiotics is not as swift as the rapid emergence of multidrug-resistant bacteria, the post-antibiotic period is imminent [3]. Therefore, the urgent need for the discovery and/or development of alternative antibacterial elements arises.

Endolysins (ELs), enzymes encoded by bacteriophages produced at the end of the phage reproduction cycle that cause lysis of the cell wall bacteria [4], are currently under increased development. Since ELs target the peptidoglycan (PG) layer, a substantially conserved component of the bacterial cell wall, no resistance problem has yet been reported with the exogenous use of these [5,6]. Hence, ELs appear as a potential alternative to conventional antibiotics.

The safety profile of ELs stands out due to their high specificity and low toxicity, characteristics that make them a promising therapeutic tool [6]. Unlike conventional antibiotics, whose broad-spectrum action can indiscriminately affect beneficial microbiota, ELs are designed to act exclusively on specific pathogenic bacteria [6,7]. This unique feature helps preserve the host’s microbiome balance and reduces the risk of dysbiosis. However, despite these advantages, it is important to consider that the selective elimination of certain bacteria could, in some cases, disrupt competitive dynamics within the microbiota, facilitating the growth of opportunistic microorganisms.

However, denoting ELs as therapeutic elements involves considerable challenges related to the way in which administration can be located in the target of infection in suitable concentrations without dissipating its activity [8]. For example, talking about strategies for EL dosing that resist the characteristic gastrointestinal tract and exert their activity in the colon represents an interesting challenge from a perspective that requires a detailed design and analysis of chemical elements that provide protection during this journey. In addition, they exert a significant effect on the colonic microbiota and help ELs in the fight against multidrug-resistant bacteria in gastrointestinal diseases. In fact, the situation in Mexico in this regard is alarming because, in 2020, the General Directorate of Epidemiology ruled gastrointestinal infections as the third most common cause of morbidity in the country, with around two and a half million reported cases [9]. Therefore, the need to carry out actions that lead to the discovery of effective and safe alternative treatments, with mechanisms of action different from those of antibiotics and with little or no risk of developing resistance, is unquestionable.

The objective of this review is not only to provide recent information about ELs applied to multidrug-resistant bacteria of clinical and nutritional interest but also to identify potential chemical elements to protect ELs from the strong conditions of the gastrointestinal tract and their possible beneficial effects by metabolites produced by colonic fermentation after their release in the colon.

## 2. Generalities of Endolysins

ELs, colloquially referred to as enzybiotics, are enzymes encoded by bacteriophages synthesized in the final stage of phage replication in the host bacteria and are able to hydrolyze the host cell wall. The bacterial cell wall presents PG as the main component, which consists of repeating monomers of N-acetylmuramic acid (MurNAc) and N-acetylglucosamine (GlcNAc), alternately linked by β (1,4) glycosidic bonds [10]. The lysis of ELs-mediated host bacteria is regulated through holins, proteins that, like ELs, are produced in the late phase of phage infection. The holins are arranged in the cytoplasm of the host, and, at a certain concentration, they oligomerize to form channels in the cell membrane for the diffusion of ELs [11]. Once diffused, the ELs manage to couple to their substrate (PG). Therefore, they hydrolyze PG via their enzymatic activity [12].

### 2.1. Structure of Endolysins

ELs that are related to Gram-positive (G+) and Gram-negative (G−) organisms are distinguished in their architecture, which is attributed to the substantial differences in the compositions of the cell walls of these two bacterial groups. In G+ ELs, two domains are known, designated as the enzymatically active domain (EAD) and the cell-binding domain (CBD) [7]. The EAD contains the enzymatic action of ELs due to the modification of specific bonds of the PG; for that matter, CBD is responsible for the folding of the enzyme to the cell wall [13]. In addition, to our knowledge, CBD increases the regioselectivity of EAD towards PG, a phenomenon commonly seen in numerous carbohydrate enzymes. In the case of G− bacteria, most ELs G− lack CBD in their globular catalytic domain structure, which is attributed to the presence of the outer membrane (OM), characteristic of this class. However, it has been reported that G− and G+ ELs may exhibit more than one CBD as well as more than one EAD. The G− KZ144 ELs of the phage *Pseudomonas* have two modular structures that contain CBD and EAD and show high affinity and broad-spectrum activity against G− pathogens such as *Pseudomonas aeruginosa* and *Bacillus subtilis*, among others [14].

### 2.2. Classification of Endolysins

**Figure 1:**
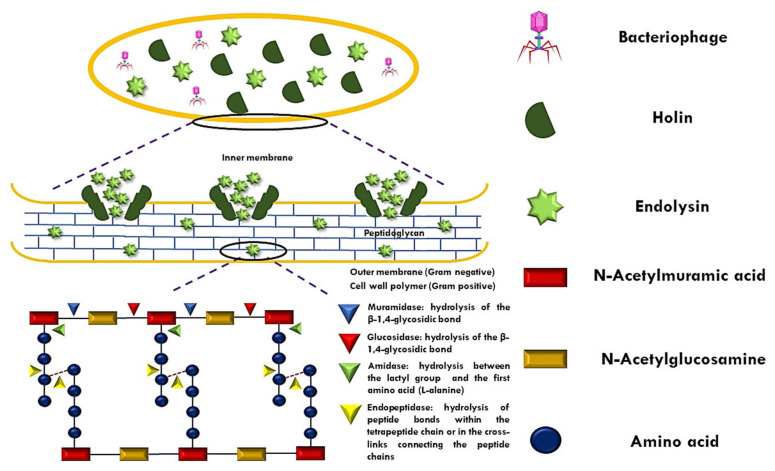
Mechanism of peptidoglycan (PG) disruption by endolysin–holin.

The classification of endolysins (ELs) is determined not only by the architecture of their amino acid domains but also by their specificity in cleaving peptidoglycan (PG). In this context, five distinct groups (I–V) have been identified and categorized based on their activity on glycosidic, amide, or peptide bonds within the PG structure [7] (Figure 1). Glucosidases hydrolyze glycosidic bonds and include the following: (I) N-acetyl-β-D-glucosamidases (EC 3.2.1.52), (II) N-acetyl-β-D-muramidases (EC 3.2.1.17, also known as “lysozymes” or muramidases), and (III) lytic transglycosylases (EC 3.2.1.17). N-acetyl-β-D-glucosaminodases target the N-acetylglucosaminyl-β-1,4-N-acetylmuramine linkage at the reducing end (GlcNAc). The other two groups break the N-acetylmuramoyl-β-1,4-N-acetylglucosamine bond; however, the transglycosylases catalyze an intramolecular reaction that results in breaking the glycosidic bond between them and forming an oxygen bridge. There is no water involved in this reaction, so lytic transglycosylases are not hydrolases, unlike lysozymes. (IV) N-acetylmuramoyl-L-alanine amidases (EC 3.5.1.28, commonly referred to as amidases) hydrolyze the amide bond between MurNAc and the first amino acid of the peptide (L-alanine). Finally, the (V) endopeptidases (EC 3.4.X.X) can be referred to as parent peptide-specific endopeptidases, in which they cut peptide bonds between two amino acids in the parent peptide, between the amino acid of the parent peptide and the amino acid at the start of the peptide–interpeptide bridge, or between two amino acids of the interpeptide bridge.

### 2.3. Endolysins and Their Conceptualization as Antibacterial

Several studies show the potential of ELs as antibacterials. Despite their late conceptualization as an antibacterial agent, numerous strengths and weaknesses have been described. Since the chemical nature of these antibacterials is proteic, it raised questions in terms of immunogenicity and stability; however, several of these concerns have been addressed, resulting in a successful preclinical outcome, and the strengths and weaknesses are summarized in Table 1. In the first part, the objective specificity is highlighted; although antibiotics exhibit a broad spectrum of inhibition once administered, they can trigger a great imbalance in the intestinal microbiota (for example, dysbiosis), particularly in commensal strains, which, as it is known until today, play a fundamental role in the health of the organism, both in the immune system and in the prevention of gastrointestinal diseases. In relation to the potential development of resistance, as a crucial point to focus on, resistance is null if the exogenous application of ELs is of a type other than that of endopeptidases, related to the fact that PG is a minimally immutable structure. It has been reported that endopeptidases, by acting on the complex crosslinking structure of PG, are mutable against various biotic and abiotic factors, so bacteria are capable of supplying one or several amino acid residues and hence undeniably affect the mode of action of this type of endolysins [13,14].

From this perspective, it is preferable to prioritize the administration of ELs from types I–IV in future research to prevent the potential emergence of resistance. Additionally, the use of “chimeric” ELs is recommended, as current studies suggest that having multiple EAD sites makes them more resistant to this phenomenon [14]. Another approach would be to employ enzymatic engineering to make specific modifications to the protein’s architecture, such as the partial replacement of N-terminal or C-terminal regions with new amino acids that impart interesting properties. Chemical elements, such as cationic or anionic peptides, could also be included to enhance their functional capabilities [26].

Furthermore, ELs can act against intracellular bacteria, though they face significant challenges due to the physical barriers imposed by the host cell membrane and the variable conditions within intracellular compartments. These bacteria are protected by the host cell membranes, which limit direct access of the ELs to the peptidoglycan. To overcome these barriers and facilitate the penetration of ELs into host cells, specific release systems have been proposed, such as the use of nanoparticles, liposomes, or the fusion of ELs with cell-penetrating peptides [26]. These strategies improve their ability to cross cellular membranes and target their objective. Finally, ELs designed for this purpose must possess specific characteristics, such as the ability to cross membranes, stability under intracellular conditions, bacterial specificity, low immunogenicity, and resistance to proteases, thereby ensuring their efficacy against these infections.

### 2.4. Individual and Combined Efficacy Against Gram-Positive and Gram-Negative Bacteria

Today, the clinical sector worldwide faces the difficult crisis of the rapid appearance and spread of bacteria resistant to one or more conventional antimicrobial agents. In fact, as far as we know, in regard to the global priority pathogens published by the WHO, nine of the twelve identified pathogens are G− [27,28] bacteria such as *Acinetobacter baumannii*, *Pseudomonas aeruginosa*, and *Salmonella* spp., to name a few. Therefore, it is necessary to expand the administration of ELs directed to G− bacteria that manage to “overcome” their characteristic barrier (OM). It is known that numerous ELs have the innate ability to diffuse through the OM; in this sense, several tactics have been proposed to facilitate these to penetrate the OM, namely outer membrane permeabilizers (OMPs), particularly organic acids as agent adjuvants; the modification of ELs by means of protein engineering; or by coupling into transport systems with OM-penetrating properties [29]. Table 2 summarizes some works in relation to the approaches used in recent years to locate ELs in G− bacteria, as well as various works related to various ELs that combat G+ bacteria.

While certain endolysins are highly effective against various Gram-negative pathogens, others exhibit activity restricted to phage strains infecting specific bacterial species. From this perspective, not only is the selectivity of inhibition by a type of bacterial genus important, but the fact of combating multidrug-resistant bacterial phyla where their inhibition undoubtedly symbolizes a transcendental fact, that is, it would be ideal to have one that is capable of inhibition in both G+ and G− bacteria. In addition, the situation of the trend based on the doses of the EL type versus another is remarkable; in other words, for modular ELs (chimeric or not), it is understood that high doses are required to achieve a potential effect against G− pathogens, a reverse phenomenon for chimeric globular ELs even without this particularity [6].

Furthermore, based on tactic four, it is undeniable that functionalization or chemical association processes substantially promote the lytic characteristics of globular ELs. Therefore, the fact that functionalization processes greatly favor the lytic characteristics of globular endolysins is unquestionable. In short, sighting interesting research projects would undoubtedly lead to generating various analyses and employing synergistic treatments in which G− chimeric ELs (non-endopeptidases) are associated with one or “n” potential chemical elements for functionalization processes in order to allow ELs to resist different adverse conditions in which their enzymatic activity is compromised and safeguard them. It is worth mentioning that most gastrointestinal diseases are associated with the level of pathogenicity of G+ bacteria (such as *Clostridium* spp. and *Salmonella* spp., to name a few), and it could be considered that it would be convenient to administer selective ELs to these genera since they have presented important results in this regard [17,18,19,20,30,31,32,33,34,35]; some even have the duality of inhibiting G+ and G− bacteria from phages that infect G+ [21,22]. Despite the above, the characterization of these ELs from the point of view of stability against abiotic factors is minimal, which, without considering the lack of analysis regarding its effect with probiotics, undoubtedly rules out its possible application even when the objective’s bacteria are key in mitigating gastrointestinal diseases. 

**Table 2:** Activity of endolysins against bacteria of clinical and nutritional interest.

Endolisyn	Description	Treatment	Activity Spectrum	Dose (µg/mL)	Specifications	References
**Strategy 1: Identification of endolysins with intrinsic OM-passing capabilities**						
LysAB2	Globular with a highly cationic α-helix in the C-terminal region	–	*A. baumannii* *E. coli* *S. aureus* (+) *S. sanguis* (+)	500	Active against G+ and G− bacteria. 2–3 log kills of bacteria in logarithmic phase (~10^6^ CFU/mL).	[36]
PlyF307	Globular and highly cationic in the C-terminal region	–	*A. baumannii*	100	More effective (~5 log kills) against log phase than stationary phase bacteria (~1.5 log kills). >5 log kills for all tests of A. baumannii clinical isolates of 10^6^ CFU/mL. 50% of infected mice were rescued from fatal bacteremia with injection of PlyF307 (1 mg). Decreased bacterial load by ~2 log.	[37]
80α, phi11, LysK	Modulate with CHAP domain and amidase	–	*Staphylococcus* spp. (+)	200	Active against a wide range of staphylococci strains and *S. aureus* biofilms.	[20]
ΦCP39O and ΦCP26F	Modulate with amidase domain	–	*C. perfringens* (+)	–	In vitro activity against various strains of *C. perfringens.*	[17]
LysPA26	Globular with a single lysozyme domain	–	*P. aeruginosa* *K. pneumonia* *A. baumannii* *E. coli*	500	2–4 log bacteria kills in log phase (~108 CFU/mL) Mejora menor con EDTA 1 y 5 mM.	[38]
KP27	Globular with carboxypeptidase activity	–	*P. aeruginosa* *K. pneumonia*	400	Possesses PG degrading activity. Not cytotoxic to human cells.	[39]
PlyE146	Globular with a muramidase activity and highly cationic C-terminal domain	–	*A. baumannii* *P. aeruginosa* *E. coli*	400	More effective (>3 log kill) against log phase bacteria (~5 × 106 CFU/mL) than stationary phase bacteria (1 log kill).	[40]
PlyGVE2CpCWB	Modulate with amidase domain and a C-terminal CBD	–	*C. perfringens* (+)	2000	Active against C. perfringens strains (~9 × 106 CFU/mL) with resistance thermometer (~50 °C).	[18]
AcLys	Globular with a C-terminal α-helix and muramidase activity	–	*A. baumannii* *P. aeruginosa* *K. pneumonia* *E. coli*	50–100	The MIC for several species of multidrug-resistant bacteria fluctuated between 50 and 100 µg.	[41]
Ply6A3	Globular with muramidase activity	–	*A. baumannii* *K. pneumonia* *E. coli* *E. faecium* (+) *S. aureus* (+)	–	*A. baumannii* more susceptible than other species Un IC_50_ de 19 µg/mL. Rescue 70% of bacteremia-infected mice with injection of Ply6A3 (2 mg) and combined therapy of Ply6A3 (1 mg) and phage PD6A3 (109 PFU/mL).	[42]
KZ144	Modulate with lytic transglycosidase activity	–	*P. putida* *P. fluorescens* *E. coli* *S. typhimurium*	–	Specificity for PG of the A1γ chemotype (G− species).	[14]
PlyLM	Modulate with amidase activity	–	*Listeria monocytogenes* (+)	–	Eliminates monolayers of *L. monocytogenes* with MIC from 158 to 744 µg/mL.	[19]
**Strategy 2: Application of OMPs and other treatments to permeabilize the OM**						
SPN9CC	Globular with transmembrane helix at the N-terminus	1–5 mM EDTA	*E. coli*	300	~2 log kills of bacteria in log phase (~106 CFU/mL). Enhanced activity (4 log kill) with PME.	[43]
GP110	Modulate with an N-terminal PBD and a C-terminal EAD.	0.5 mM EDTA	*P. aeruginosa* *S. typhimurium*	72.5	~2 log phase bacteria kills 106 CFU/mL.	[44]
Ply17	Modulate with an N-terminal PBD and a C-terminal EAD	0.1–5 mM EDTA	*P. aeruginosa* *E. coli*	1000	>3 log removal in log phase (108 CFU/mL) with 0.5 mM EDTA.	[45]
LysB4	Modulate with endopeptidase activity	0.1 M EDTA	–	5	Broad spectrum of inhibition against G+ bacteria (*L. monocytogenes*, *B. cereus*, *B. subtilis*) and G– (*S. Typhimurium*, *E. coli*).	[21]
Lys394	Globular with muramidase activity	Poly-L-arginine (5–15 kDa) 0–1 mM EDTA PGLa peptide	*E. coli*	–	The use of OMPs caused the lysis of planktonic bacteria in a dose-dependent manner.	[46]
LysABP-01	Globular with muramidase activity	Colistin	*A. baumannii* *P. aeruginosa* *E. coli*	500	Synergistic interaction with colistin.	[47]
ABgp46	Globular with acetylmuramidase activity	Citric acid (3.65 mM) Malic acid (4.55 mM) EDTA (0.5 mM)	*A. baumannii* *P. aeruginosa* *S. typhimurium*	46.2	Intrinsically active (2 log kill) against *A. baumannii* log phase culture (106 CFU/mL). >5 log kills of A. baumannii and 4 log kill of other G− bacteria with OMPs.	[48]
Lysep3	Globular with lysozyme activity	EDTA 25 mM	*P. aeruginosa* *E. coli*	–	Active against log phase culture (10^6^ UFC/mL)	[22]
**Strategy 3: Endolysin design to promote endolysin uptake through the OM**						
Cpl-7S	15th amino acid substituted in CBD for Cpl-7 derived from pneumococcal phage Cp-7	0.01% carvacrol	*S. pneumonia* (+) *S. pyogenes* (+) *E. coli* *P. putida*	5	3 log kills of *E. coli* in log phase (107 CFU/mL) in the presence of carvacrol. Increased survival rate of zebrafish infected with *S. pneumoniae* or *S. pyogenes*.	[49]
Artilisyn	Fusing PCNP at the N-terminus to two modular lysines (OBPgp279 and PVP-SE1gp146)	0.5 mM EDTA	*P. aeruginosa* *A. baumannii* *E. coli*	53.3	Increased activity due to modification in OBPgp279 and PVP-SE1gp146. 3% survival rate in a *C. elegans* model (*P. aeruginosa* PA14).	[50]
**Strategy 4: Application of endolysins in carrier systems**						
4Lyz-CBM	Fusion of a cellulose-binding module (CBM) to globular T4Lyz with amphipathic α-helix and muramidase activity	Pretreated with chloroform	*E. Coli* *P. mendocina* *M. lysodeikticus* (+)	200	4 log and 1.3 log deaths of *E. coli* and *P. mendocina*, respectively No loss of activity after immobilization of the fused protein.	[51]
BSP16Lys	Cationically charged BSP16Lys encapsulated liposome composed of DPPC, cholesterol, and hexadecylamine	–	*S. typhimurium* *E. Coli*	94.5	No native endolysin activity without PME Encapsulated endolysin had a 2.2 log and 1.6 log kills against *S. Typhimurium* and *E. coli*, respectively, at ~103 CFU/mL.	[52]

CBD, cell-binding domain; MIC, minimum inhibitory concentration; DPPC, dipalmitoylphosphatidylcholine; EAD, enzymatically active domain; EDTA, ethylenediaminetetraacetic acid; IC_50_, mean inhibitory concentration; OM, outer membrane; PBD, PG-binding domain; PCNP, polycationic nanopeptide; PG, peptidoglycan; PME, outer membrane permeabilizer; PFU, plate-forming units.

### 2.5. Effect of Abiotic Factors on Endolysins

The stability of ELs during their collection, storage, and administration is essential. Table 3 summarizes some of these antibacterials from preclinical studies, where various aspects of the stability of endolysin G− such as temperature, catalytic activity against pH, and storage specifications are considered. After their rapid discovery by genetic engineering and promising results in the microbiological area, undoubtedly, the lines of research in which these antibacterials can be applied are increasing day by day, which, based on this, merits being pointed out in one or several stability aspects.

First, when theorizing a scenario of a potential application at the industrial level, not only does thermostability play an important role to consider, but the pH does as well. Emphasizing the problems raised initially, if an application associated with exerting a substantial effect on the intestinal microbiota is glimpsed, the pH parameter would be of vital interest since the important changes of this abiotic factor in the gastrointestinal tract are characteristic. In addition, the optimal pH should be close to neutral since, as far as we know, this is the usual pH of the colon in basal conditions, although, in reality, the colon is in constant activity as a result of colonic fermentation, which can lead to a considerable decrease in pH as a result of the generation of short-chain fatty acids (SCFAs) [53]. Therefore, the ELs to be applied must exhibit catalytic activity in a wide pH range. Finally, both in the industrial sector and in the field of science, the scenario “reserved for later analysis” is common, in such a way that storage appears as a crucial item to consider due to the fact that ELs may lose effectiveness.

In general, and based on what is reported in the literature, the endolysins KZ144 and LysPA26 would be ideal candidates as antibacterial agents since the three aspects addressed in this section reflect a solid enzymatic activity in addition to exhibiting a significant inhibition spectrum against bacteria of clinical and nutritional importance [13,24].

### 2.6. Commercial Endolysins

Today, there are pharmaceutical products with ELs as an active ingredient, mainly aimed at treating diseases and their respective symptoms resulting from pathogenic bacterial proliferation, particularly infections caused by staphylococci; the companies and their respective products are listed in Table 4. It is noteworthy that, although these products have performed well in the market, there is still a narrow path and an apparent gap in relation to products aimed at the treatment of gastrointestinal infections. Studies on ELs for gastrointestinal infections are limited due to several factors. The acidic environment of the stomach and the intestinal barriers hinder the penetration and stability of ELs. Moreover, the complexity of gastrointestinal infections, which involve a diverse microbiota and a much more regulated environment, presents additional challenges for the development of endolysin-based therapies. The lack of research in this area may also be related to the absence of suitable models to study the dynamics between ELs and the intestinal microbiota, as well as other phenomena specific to the digestive tract. These models are not yet sufficiently developed to accurately simulate the interaction between the enzymes and the conditions of the intestinal environment. The need to develop more advanced methods for administering ELs effectively in the digestive tract also contributes to the limited research in this field.

Therefore, although their administration implies a palliative effect, they will serve as a starting point to venture into the design, evaluation, and application of ELs and their association with adjuvant chemical elements, thus initiating the era of “biocomposites”.

## 3. Biocomposites and Colonic Fermentation: The Key to Combating Gastrointestinal Diseases

### 3.1. Definition of Biocomposite and Its Potential Antimicrobial Activity

In a chemical sense, a biocomposite is a material system made up of the combination of two or more chemical entities that differ in form and chemical composition and are substantially insoluble with each other. Additionally, strictly speaking, each element that makes it up is organic in nature; its design is normally linked to obtaining a composite material with interesting biological, physicochemical, and/or technofunctional properties, and since the chemical elements are isolated, these characteristics are difficult to achieve [54]. Nowadays, biocomposites have been used for a long time in the health area since they mostly exhibit antimicrobial activity; in fact, they have a certain desirability because they pose a minimal or even zero risk of developing resistance [55]. For this reason, the field of antimicrobial biocomposites has experienced constant development in recent years and is known to be increasingly relevant during the current COVID-19 pandemic. In this sense, Table 5 shows some of the current works on antimicrobial biocomposites.

Ideally, any biocomposite linked in the health sector for its administration as an antimicrobial agent should meet certain characteristics such as biocompatibility, bioavailability, and safety after its application and/or ingestion [61]. The usual strategies that are considered for the development of an antimicrobial biocomposite have been associated with the use of biopolymers with innate antimicrobial capacity or the addition of organic compounds in the polymeric matrix [62]. In this sense, the potential therapeutic target in the context of this review is unquestionably the colon. Therefore, in addition to considering the arguments mentioned above, these must necessarily present little or no bioaccessibility and, furthermore, it would be interesting if they exhibit a carbohydrate-type nature, specifically non-starchy, attributed to the fact that these have already been reported as a potential substrate for the beneficial bacteria of the colon and hence reflect an antimicrobial effect based on the concept of a “prebiotic effect” [53].

### 3.2. Colonic Fermentation and Gastrointestinal Health

The colon, an organ of vital importance in human health, is a complex ecosystem in which the microbiota encompasses various metabolic niches. Bacterial counts in intestinal contents gradually increase from the proximal colon to the distal large intestine, and to our knowledge, viable cell counts in fecal matter fluctuate between 10^11^ and 10^12^, where the final stages of the digestive process are mediated by colonic microorganisms [63]. Today, it is known that their composition turns out to be multifactorial, namely based on host (genetics, diet, disease, use of drugs and antibiotics), microbiological (competition for nutrients and adhesion sites, cooperative metabolism, bacterial antagonism), and environmental (availability of substrate, local pH) factors [63]. Intestinal bacteria produce hydrolytic enzymes that mainly digest complex dietary carbohydrates (not absorbed in the intestine) and consequently contribute significantly to host metabolism through so-called colonic fermentation. From this perspective, the complexity of different carbon sources to which microorganisms have access is the primary regulator of microbial diversity in the colon, where intestinal microorganisms mostly prefer saccharolytic compounds, particularly non-digestible starchy compounds. These include non-digestible oligosaccharides such as fructooligosaccharides) (FOSs), galactooligosaccharides (GOSs), isomaltooligosaccharides (IMO), xylooligosaccharides (XOSs), raffinose, resistant starches, and plant cell wall polysaccharides (dietary fiber) [64].

To our knowledge, carbohydrate fermentation gives rise to short-chain fatty acids (SCFAs), particularly acetic acid (AA), propionic acid (PA), and butyric acid (BA), known as the main products of fermentation, and through their absorption and metabolism, the host can obtain energy from the part of the food that is not digested [65]. SCFAs present a set of effects in the body and affect the transport and metabolism of epithelial cells, as shown in Table 6. Of note, the most important interactions between the colonic microbiota and the gut are associated with the metabolic effects of SCFAs on colonic epithelial cells. Probably the most important interactions between colonic and intestinal microorganisms can be attributed to the metabolic effects of SCFAs on colonic epithelial cells [65]. AA, PA, and BA are oxidized by mucosal cells to supply energy. However, BA is of particular interest from this perspective because of the various physiological effects that have been reported so far. Epithelial cells in the distal colon derive 60–70% of their energy needs from bacterial fermentation, and in terms of their metabolic importance, the priority is BA > PA > AA [66]. While colonocytes also oxidize glucose and glutamine, more than 70% of the oxygen consumption can be attributed to the oxidation of butyrate [66]. Due to the above and based on what is reported in the literature, the relevance of SCFAs (especially BA) in the health of the host, specifically, is undeniable in a healthy state of the individual.

## 4. Biocomposites: Novel Strategy for the Administration of Endolysins

The isolation and application of bioactive compounds or nutraceuticals, once a significant challenge, have become a prominent area of research. A major limitation, however, is their low bioavailability during digestion due to interactions with the food matrix and external factors. To address this, recent advancements have focused on embedding these compounds into biomaterials, a strategy that enhances their stability, controlled release, and functional efficacy. Due to the above, the generation of biocomposites has symbolized a competent tactic in the preservation of the functionality of these compounds, attributed to the fact that a prolonged release has been achieved, and hence, they are able to exert their biological activity on the therapeutic target. In terms of biocomposites of a protein nature, interesting results have been achieved with lactase [90], papain [91], and zein [92], to name a few. However, it is crucial to determine the polymeric material(s) to be used, which, in addition to contributing to ELs’ stability, would be interesting if they contributed significantly to the events discussed above.

### 4.1. Alginate Oligosaccharides as an Important Wall Element

Alginate oligosaccharides (AOSs) are known as those that are linked by means of 1,4-glycosidic bonds with a degree of polymerization of 2–8 and have mannuronic acid (M) and guluronic acid (G) as base monomers, achieved by the breakdown of alginate by means of physical, chemical, or enzymatic methods where these residues form homopolymeric segments in the molecule, such as polymannuronic acid formed by M units with β-D-(1,4) bonds, the formation of polyguluronate connected to α-L-(1,4) by G units, or three structural fragments of segments alternately copolymerized with M and G [34,35,36]. It would be interesting to consider AOSs since, although their study in various fields has been exploited in the last twenty years, it has not led to key works on the formation of biocomposites [93,94].

The formulation of a product containing these compounds presents a viable and promising opportunity, given their diverse biological activities, including antioxidant properties [37,38,39], prebiotic effects [40,41,42], immunomodulatory functions [43,44], and antibacterial activity against clinically and food-relevant bacteria [95], as well as aquaculture pathogens such as *Vibrio parahaemolyticus* and *Vibrio harveyi*. Notably, vibriosis and other resistant pathogens responsible for gastrointestinal illnesses pose a significant public health threat, particularly due to the consumption of contaminated shellfish. For instance, in the United States alone, the number of diagnosed infections caused by *Vibrios* increased by 311% in 2018 compared to 2015–2017 [96]. Regarding the prebiotic effect, it is worth emphasizing that alginate oligosaccharides (AOSs) demonstrate a superior prebiotic effect compared to fructooligosaccharides (FOSs) [97] and xylooligosaccharides (XOSs) [98]. This is evident in their greater capacity to promote the production of SCFAs and enhance the growth of beneficial bacteria while inhibiting pathogenic bacteria, highlighting their potential for therapeutic and nutritional applications.

So far, the reason for this phenomenon has not been elucidated; however, the hypothesis that we propose in this regard is described below. Among the effects produced in the colon, it is worth mentioning that a prebiotic effect is characteristic when the growth of beneficial fermentative bacteria (*Bifidobacterium* spp. and *Lactobacillus* spp.) is stimulated by generating SCFAs that produce a considerable drop in pH, modulating the development of certain communities that can be associated with harmful effects (*Bacteroides*, *Fusobacterium*, *Salmonella* spp. and *Clostridium* spp.). *Bifidobacteria* do not produce BA but stimulate the growth of SCFA-producing bacteria in the colon. In fact, several species of colonic bacteria are known to produce BA from lactic acid (LA), and this could stimulate BA formation indirectly through the cross-feeding of metabolites. Due to the above, and taking into consideration that the aforementioned pathogenic microorganisms are mostly neutrophils, undoubtedly, the pH at the beginning and during colonic fermentation is the medullar point.

Although there are innumerable works that mention sources of prebiotics and their effect on colonic fermentation, none of them describe or even mention the relevance of the chemical nature of the non-starch carbohydrate throughout this phenomenon. In this sense, it becomes interesting to approach this event from the perspective that the prebiotic is “accelerating” the colonic fermentation process, which reflects the capacity of this substrate per se to significantly reduce the pH. AOSs, for example, unlike FOSs, GOSs, XOSs, and IMO, G, and M, exhibit an acid moiety due to the presence of the carboxyl group (-COOH), which, being in a colonic medium, can initially lower the pH considerably and exert at a given time a bacteriostatic effect on pathogenic bacteria. In addition, once significant concentrations of AA, PA, BA, and LA are produced, they promote a highly acidic pH more quickly due to the accelerated dissociation of the carboxyl group based on their acid dissociation constants (pKas), which, for G and M, are 3.65 and 3.38, respectively [99]. In fact, a synergistic effect could be clearly reflected since as colonic fermentation progresses, not only would SCFAs and organic acids acidify the medium, but AOSs would contribute to said phenomenon and therefore the inhibition of pathogenic bacteria associated with diseases.

Gastrointestinal cells would die, even without taking into account the generation of extracellular products and competition for substrate and space. On the other hand, there are factors related to the probability of success regarding the fact that the previously mentioned effects are exhibited by AOSs, which are directly related to the chemical structure, particularly the ratio or quotient of M and G (M/G). It is known that each monomer is intrinsically linked to a particular characteristic, that is, a greater number of blocks of M than G is said to exhibit a better bioactive profile; on the contrary, it correlates with high stability against abiotic factors [37,39,40,44,45,49,50]. Then, it is stated that if the objective of the treatment is for AOSs to significantly exhibit the described biological effects, then M/G > 1, and if the objective is to show significant stability against an abiotic factor (for example, pH), then M/G < 1, and finally, when hypothesizing an event where both phenomena are manifested as much as possible, then M/G ≈ 1. Taking into account the double problem raised previously in relation to the application of ELs, it would be unquestionably recommended to opt for the last conditioning factor.

**Figure 2:**
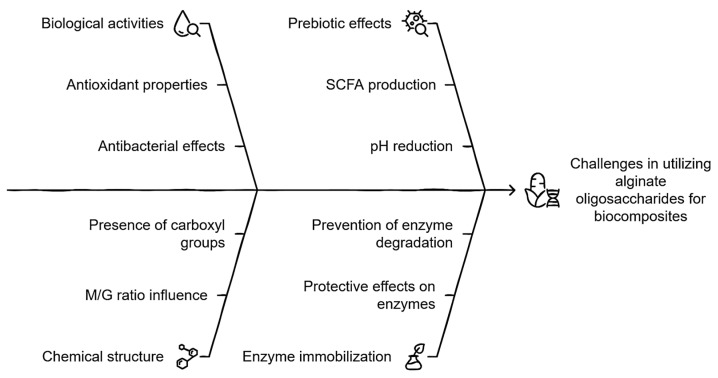
Integral schematization of the role of AOSs in the development of biocomposites.

Finally, in relation to questioning whether AOSs are really ideal elements to form a biocomposite together with ELs, there are already recent works that approve this situation since excellent results have been obtained in the immobilization of enzymes with AOSs [33,51], attributed to the fact that AOSs can exert a protective effect on the enzyme by preventing its rapid degradation. Figure 2 integrates the main concepts associated with biocomposite development described above, paying special attention to the role of AOSs as a central element in its formulation.

### 4.2. Endolysin Carrier Potential: Modified Cellulose

Cellulose (CL) is the most abundant biopolymer on Earth; it constitutes all the cell walls of plants and occurs in highly ordered chains of β-(1,4) glucans that are produced naturally or through chemical processes. From the point of view of the generation of biocomposites, CL is a material that has biocompatibility, bioaccessibility, flexibility, formability, non-toxicity, and mechanical resistance with a porous nanostructure [100]. It is known that the large number of hydroxyl groups present in the anhydrous glucose that make up CL are specific reactive groups that can bind to compounds capable of giving this biopolymer new characteristics. Therefore, many researchers have made efforts to conjugate different molecules in CL to change their attributes. In principle, it is noteworthy that CL today is strongly used for the generation of biocomposites of both bioactive compounds and protein products [53,54,55]. However, it is a challenge to determine the spacer group that will act as a “bridge” between ELs and CL, where there are currently numerous methods to achieve it [61], although the use of chlorinated compounds (particularly acyl chlorides) should be highlighted since in addition to causing the hydroxyl group to go through deprotonation (the ideal event to form a covalent bond with a group characteristic of ELs, for example, the amino group), it has also been shown that the reaction conditions are not strong and do not produce aberrant reactive species [61].

**Figure 3:**
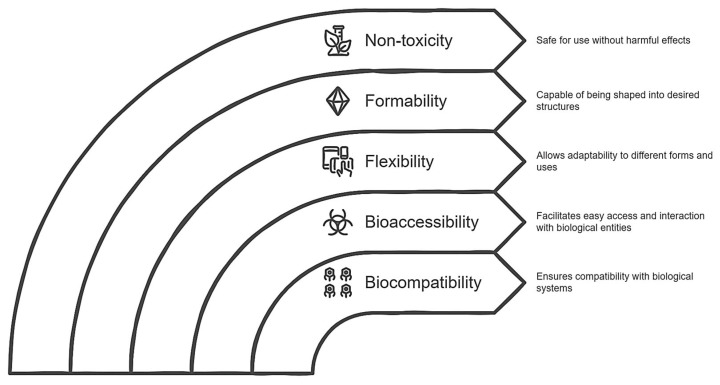
Diagram of the role of modified cellulose as a potential carrier agent for an endolysin.

The fact of incorporating modified CL involves two eventualities: first, it is known that CL does not have fermentation capacity due to its structural complexity (intramolecular bonds) [65], and second, the modification of CL (acylation) can lead to some adverse event in terms of colonic fermentation. For the first case, as far as we know, there are reports that CL, when coupled with some non-starch carbohydrate (for example, xyloglucans), manages to potentiate the fermentative capacity reflected in a significant increase in SCFAs [101], possibly estimated in this proposal. Finally, it is an atypical situation for a potentially fermentable compound to have the acyl group in its chemical structure since this is normally related to lipophilic compounds that are absorbed in the small intestine, so there are few reports regarding the pathway of bioconversion of this group or if the colonic metabolites may be harmful to the health of the host. From this perspective, the only precedent in this context argues that the acyl groups do not compromise the host colon at all since aberrant metabolites are not produced, and even the SCFA profile is favored. In fact, despite not elucidating a possible bioconversion mechanism, it is hypothesized that the colonic microbiota is capable, through lipases and/or transferases, of releasing alkyl groups to give way to methylations or else, a process that helps in the coupling of SCFAs with secondary metabolites of poor bioaccessibility, a phenomenon commonly appreciated in colonic fermentation (particularly phenolic compounds) [57,58,59] such that even, from a metabolic point of view, bioenergetically, this phenomenon is preferable for the colonic microbiota. As discussed, Figure 3 summarizes the key points of biocomposite development, emphasizing the role modified CL plays in the connection between theoretical design and its practical application.

### 4.3. Formation of the Biocomposite: Endolysin to Choose

Finally, determining that ELs could have remarkable stability in the colonic medium represents a crucial point in the formulation of the biocomposite. In our opinion, ELs that have eminent activity in conditions similar to those of the colon should be selected as a study model. Therefore, the acceptance criteria would be exhibiting activity in a wide pH range, particularly in acid conditions (pH 4–7, with optimal activity 2–7), exhibiting a tolerance against divalent cations (Mg^+2^ and Ca^+2^), and showing evidence of bactericidal activity against G− pathogens (*Vibrio* spp. falls into this category) and effectiveness against G+ pathogens other than probiotic genera (such as *Bifidobacterium* spp. and *Lactobacillus* spp.). Based on these requirements, the ELs KZ144 [14] and LysPA26 [38] would be the ideal candidates. Emphasizing KZ144, it is known that it maintains up to 80% of its activity when exposed to concentrated solutions of divalent cations (Mg^+2^ and Ca^+2^), it has a substantial bactericidal effect with G− and G+ bacteria (demonstrating ineffectiveness with probiotics), and its optimal pH range is ideal for colonic fermentation initiation conditions [14]. In short, the EL KZ144 would be selected to form part of the biocomposite and, to a lesser extent, LysPA26 could as well. Although both come from phages that infect G− bacteria, they differ in relation to the type and amount of EAD and CBD. It is logical to carry out various analyses to elucidate the relevance of these characteristics in this context.

Biocomposites offer a promising advantage over traditional antibacterial methods due to their high specificity and effectiveness in lysing bacterial cells, especially within biofilms. Unlike conventional antibiotics, they carry a lower risk of inducing bacterial resistance. Moreover, biocomposites can be modified or combined with other treatments to enhance their antimicrobial properties, positioning them as a potential solution for combating multidrug-resistant bacteria.

It is crucial to highlight the importance of their conjugation with non-starch saccharides, not only as carriers but also for their potential synergistic role in disintegrating the matrix in the intestine. This theory could be validated in vitro and later in vivo, offering an innovative perspective. Although there is evidence of the antibacterial effect of endolysins, reports on their conjugation with stabilizing agents are scarce, especially in cases like KZ144 and LysPA26.

**Figure 4:**
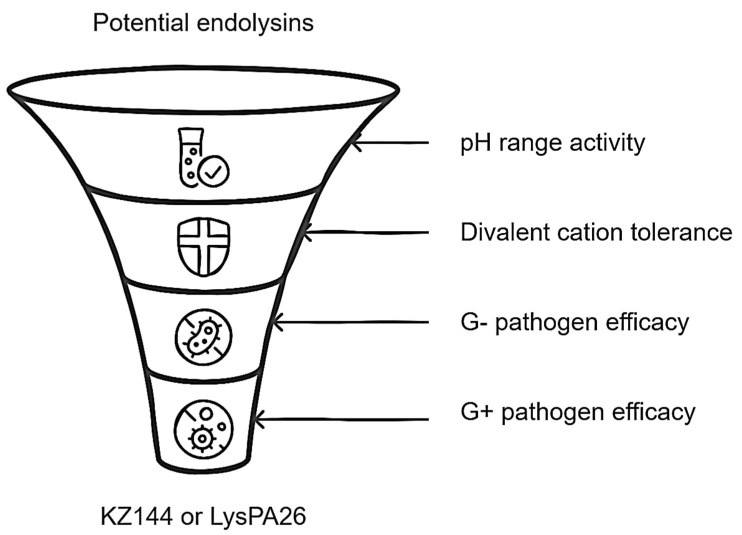
Relevant features for endolysin selection in biocomposite design.

According to the above, the scheme presented in Figure 4 summarizes the main points associated with the development of the biocomposite, emphasizing the role of endolysin to be loaded.

**Figure 5:**
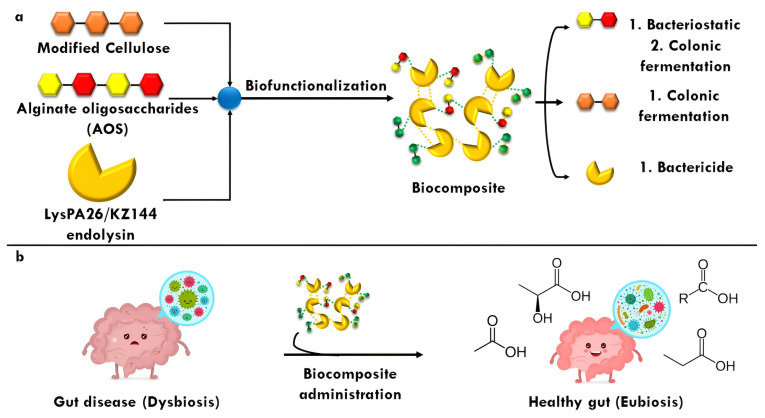
Synthesis of biocomposite from alginate oligosaccharides (AOSs) and modified cellulose (CL) embedded to an endolysin (EL). (**a**), the released components could exert antibacterial and postbiotic effects; (**b**), a dysbiosis caused by a gut disease could be mitigated by the biocomposite administration.

In summary, the imperative need to provide protection to endolysins is unquestionable since their enzymatic activity could be compromised by biotic and abiotic factors such as the complex gastrointestinal tract. Therefore, the use of polymeric materials such as modified CL and AOSs would be excellent candidates to be biofunctionalized with these enzymes and give way to the formation of the biocomposite since they chemically represent suitable molecules to be loaded and show an extensive bioactive profile, respectively. Finally, ensuring that the ELs to be biofunctionalized are stable throughout colonic fermentation is prioritized, and, based on their chemical composition among other characteristics, the EL KZ144 would be the ideal in this context, with LysPA26 as a second option (Figure 5).

**Figure 6:**
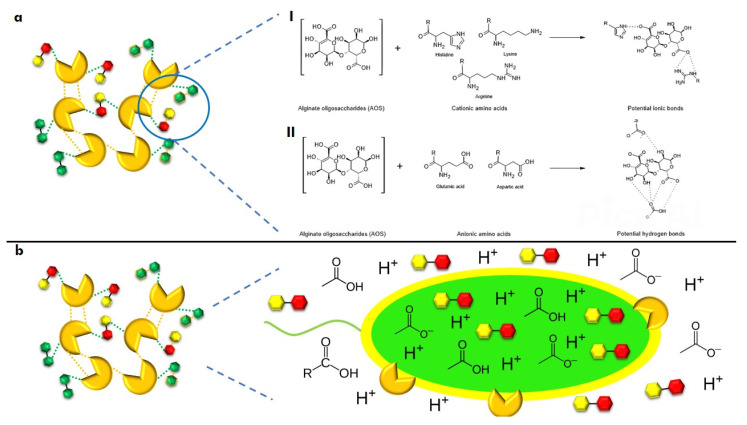
Potential chemical interactions of biocomposites and their impact on bacterial cell death. (**a**), interactions between components through ionic bonds (I) and hydrogen bonds (II); (**b**), effect of the components on bacterial cell death through intra and extracellular pH modification.

Finally, potential interactions can be hypothesized by considering the chemical characteristics of the biocomposite components and their potential effect on cell death (Figure 6). The G and M groups of AOSs have pKas of 3.65 and 3.38, respectively, suggesting that the biocomposite should be synthesized under alkaline conditions. Both LysPA26 and KZ144 are active in basic environments [13,24], indicating that conjugation with AOSs would occur spontaneously [92]. The acylated portion of CL could facilitate passive diffusion into the bacterial cell, allowing free hydroxyl groups to interact with AOSs and CL.

We hypothesize that endolysins (ELs) could neutralize the bacterial cells’ surface charge, reducing repulsive forces and allowing for greater embedding. Furthermore, under alkaline conditions, a balance of the protonation/deprotonation of the ionizable groups of amino acids and diaminocarboxylic acids could be achieved, enhancing chemical interactions within the biocomposite, such as ionic bonds (Lys or Arg with carboxylic acid groups) and hydrogen bonds (Glu or Asp with carboxylic acid groups) (Figure 6a).

At the intestinal level, it is theorized that AOSs could generate SCFAs [30,42], which pathogenic bacteria would use as a carbon source, leading to energy depletion. This effect could be enhanced by the non-metabolized acidic fraction. Synergistically, if the biocomposite enters the intracellular space, AOSs could induce cell death through excessive ATP production while endolysins activate their previously described mechanisms of action (Figure 6b).

## 5. Current Trends of Applied Endolysins for Gastrointestinal Diseases

In any investigation, the retrieved results are regularly explored, described, and organized. Efficient and affordable technologies capable of grouping the retrieval and subsequent organization of information are currently used. In this sense, the grouping of the results in the so-called clusters has represented an interesting approach. The Carrot2 program performs the “clustering” of the reported works based on similarities between them. The user develops some general themes and can then analyze the more specific themes set dynamically from the query results. Due to the above, a stratified search [102] was generated to glimpse the current trends of the relevant contents of this review, which was divided into two important points: the use of ELs as a treatment for gastrointestinal diseases and biocomposites directed at G+ and G− bacteria of clinical and nutritional interest using keywords and boolean operators [103], The expressions “endolysin AND gut disease AND treatment NOT antiobiotics” and “ biocomposite OR composite AND antibacterial AND clinical OR nutritional” were used, respectively.

**Figure 7:**
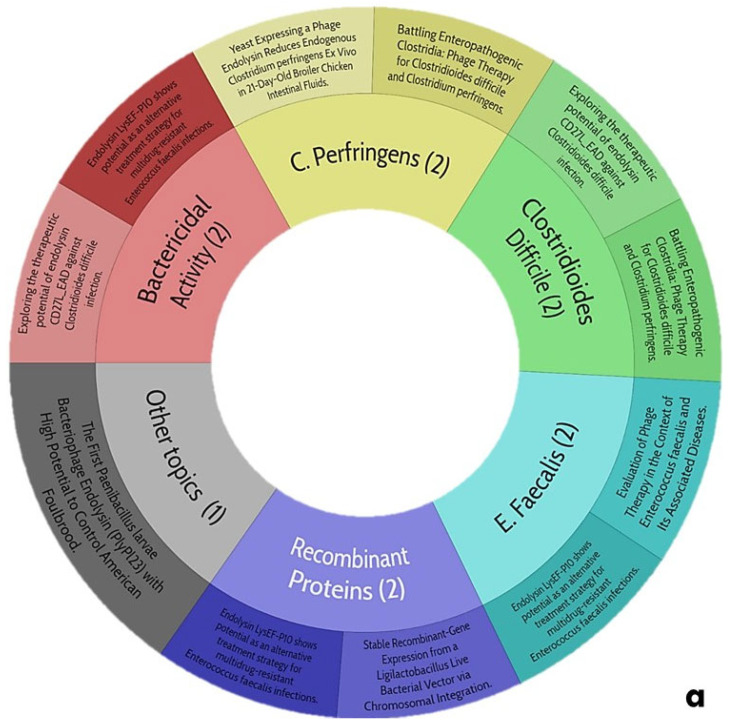

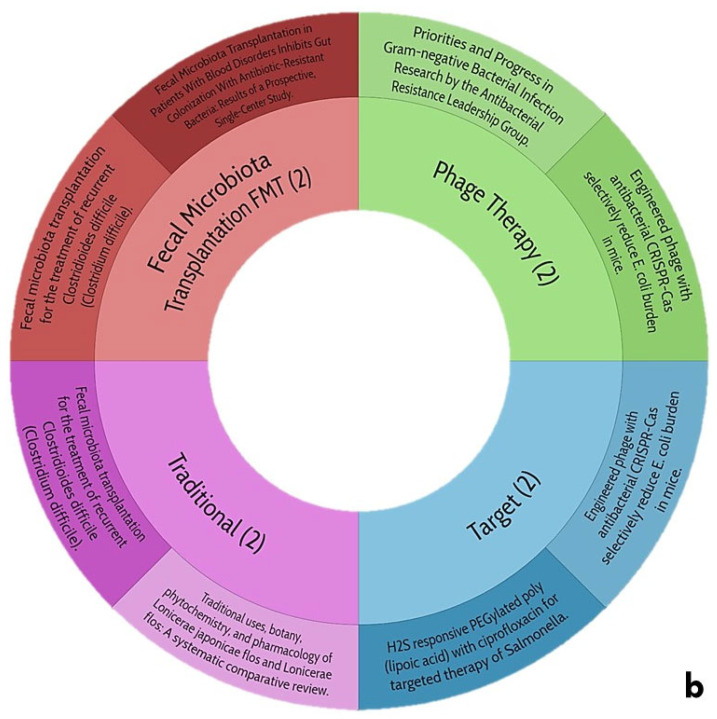
Trends of central themes of this review: (**a**), use of endolysins as treatment for gastrointestinal diseases; (**b**), biocomposites directed at Gram-positive and negative bacteria of clinical and nutritional interest.

The information obtained that exposes the main topics of this work is shown in Figure 7.

### 5.1. Current Trends in the Use of Endolysins as Treatment for Gastrointestinal Diseases

The potential trends in the use of ELs as a treatment for gastrointestinal diseases (Figure 7a) are winding since the search yields only one work that talks about the characterization of an EL with significant activity against *Clostridioides difficile*, which is notable for its range of pH and thermostability, as it could be a candidate for the proposal of this review. However, the authors mention that it exerts an inhibitory activity against prebiotic bacteria (*Lactobacillus* spp.) In addition, they do not report the effect of divalent cations in this EL; for this reason, it seems risky to consider it. Despite this, there is no scientific evidence of any ELs subjected to a gastrointestinal process neither in vitro nor in vivo since the pathogenic microbiota lives in the colon, and therefore, it is imperative to evaluate this situation. For this reason, it is reiterated again that the proposal of this work will be innovative and will open the door to future research in this field.

### 5.2. Current Trends in Biocomposites Directed at Gram-Positive and -Negative Bacteria of Clinical and Nutritional Interest

When generating a stratified exploration for biocomposites directed to G+ and G− bacteria of clinical and nutritional interest (Figure 7b), the data are limited, a trend is observed with respect to the exacerbated use of biopolymers aimed at being biofunctionalized and subsequently being evaluated under a criterion at the biological level, where modified CL has been widely used in this situation, and it is to be expected since it is an affordable element, in addition to the fact that its manipulation at the chemical level is practical. Unfortunately, there is no work that speaks about any antibacterial biocomposite and even less about its application against any clinical picture regarding any gastrointestinal disease. This search for information is central to the proposal in question and provides the basis and support for venturing into the generation of antimicrobial biocomposites aimed at treating gastrointestinal diseases to curb, as much as possible, the over-administration of antibiotics helping to mitigate the spread of multidrug-resistant bacteria and truly reaching the post-antibiotic era.

## 6. Challenges and Opportunities in the Application of Antibacterial Biocomposites for Gastrointestinal Diseases

Despite advances in antibacterial biocomposites, there is still a lack of studies focused on their application to treat gastrointestinal diseases. One of the main challenges is the dynamic and regulated environment of the gastrointestinal tract, where the acidic conditions of the stomach, digestive enzymes, and barriers such as the intestinal mucosa make it difficult for many biocomposites, especially those based on enzymes like ELs, to maintain stability and effectiveness. These biocomposites must be resistant to these factors in order to ensure their functionality.

In this sense, a relevant question in the context of this review would be the following: in a state of gastrointestinal disease that substantially compromises the health of the individual that would be pertinent, should the individual be administered antibiotics that he knows will end the disease in a few days with the risk of contributing to the resistance of bacteria to these chemical substances coupled with a potential dysbiosis? Could the individual choose to ingest a biocomposite formulated with LE and non-starch poly/olisaccharides that are known not to exhibit a phenomenon of resistance after its application and SCFA production contribute to combating pathogenic colonic microbiota, respectively? Due to the foregoing, added to the fact that there are no works on any treatment to combat gastrointestinal infections that have ELs as an active ingredient, it is undoubtedly interesting and attractive to venture into the design of antimicrobial biocomposites and give guidelines for future research regarding the development of this new class of composite materials.

Unlike traditional antibiotics, which often disrupt the balance of microbial communities by indiscriminately eliminating both harmful and beneficial bacteria, ELs are specifically designed to target pathogenic bacteria while preserving the integrity of the beneficial microbiota. This characteristic makes them an appealing alternative or complementary option to conventional antimicrobial strategies, particularly in applications where maintaining microbiome balance is critical for overall health. Additionally, the incorporation of AOSs in therapeutic approaches offers significant benefits, particularly in promoting gut health. Alginate oligosaccharides are known to be metabolized by certain gut bacteria, leading to the production of SCFAs. The combination of ELs and alginate oligosaccharides thus represents a promising strategy that not only precisely targets pathogenic bacteria but also supports the growth of beneficial bacteria, resulting in a synergistic effect. This dual action could enhance therapeutic outcomes while minimizing disruptions to the microbiota. However, further research is needed to explore the interactions between ELs, AOSs, and the microbiota, especially under different physiological conditions. Understanding these dynamics will be crucial for optimizing the efficacy and safety of these innovative therapeutic approaches.

Thus, another key challenge is the interaction with the intestinal microbiota, which plays a critical role in digestive regulation and protection against pathogens. Biocomposites must be pathogen-specific without negatively affecting the beneficial microbiota. The lack of appropriate models simulating this dynamic limits the ability to predict the long-term effects of these compounds, which could lead to unwanted risks. At the formulation level, it is crucial that biocomposites can cross biological barriers and reach infection sites. The use of controlled release systems may be an effective strategy, but these technologies are still in development and require optimization to ensure clinical efficacy and safety.

**Figure 8:**
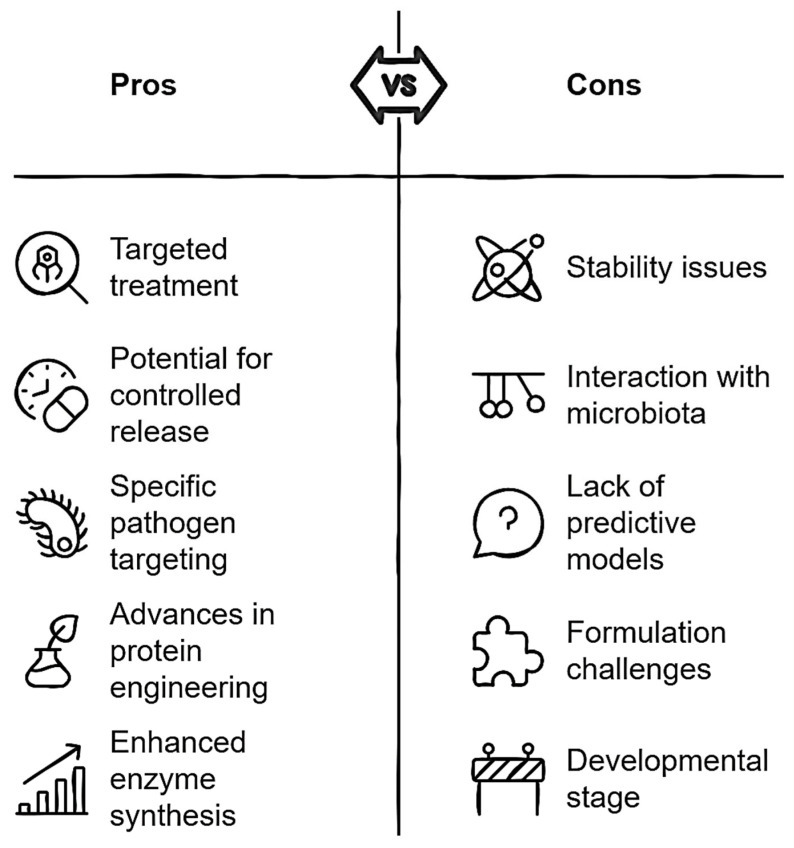
Critical analysis of antibacterial biocomposites for gastrointestinal disease applications.

To advance the application of biocomposites in gastrointestinal diseases, it is necessary to develop more accurate models that simulate the interaction between biocomposites and the intestinal microbiota. Additionally, studies should focus on engineering biocomposites that are resistant to the conditions of the gastrointestinal tract, including acidic pH and digestive enzymes, as well as improving controlled release capabilities. It is also important to enhance bacterial specificity, ensuring that biocomposites are effective against specific pathogens without harming the intestinal microbiota. Lastly, advances in protein engineering and the synthesis of enhanced enzymes could offer new opportunities to design more robust and effective biocomposites. According to the above, the scheme shown in Figure 8 summarizes the advantages and disadvantages of the application of biocomposites in the control of gastrointestinal diseases.

## 7. Conclusions

To address the growing issue of antibiotic resistance, research has increasingly focused on the development of new antimicrobial agents. In this context, ELs are gaining attention due to their potential effectiveness against drug-resistant bacteria. However, a key challenge for their implementation is the characteristic OM of Gram-negative bacteria, which restricts ELs from accessing peptidoglycan substrates, thereby reducing their efficacy. Despite this, several approaches have been proposed to facilitate OM permeabilization, enabling ELs to exert their antimicrobial activity more effectively. In recent years, various strategies have been synthesized that support the use of globular ELs as competent antibacterial agents. Although more in vivo studies are needed to better understand the safety and distribution profiles of ELs, the design of biocomposites represents a promising strategy to enhance the efficacy and stability of these molecules. It is essential to consider that the molecule to be loaded must possess a particular chemical structure and exhibit a significant bioactive profile to be effective in this process. In this regard, we have reviewed the candidate molecules for this treatment, highlighting the relevant role of modified CL and AOSs in the fight against drug-resistant bacteria. The success of a palliative treatment depends not only on the lytic activity of the ELs but also on the metabolites produced by the colonic fermentation of the materials that make up the biocomposite. Specifically, non-starch carbohydrates such as AOSs and modified CL have the potential to generate SCFAs, which could contribute to a significant prebiotic effect. This synergistic effect between ELs and the prebiotic compounds of AOSs could play a crucial role in enhancing the eradication of drug-resistant bacteria that could colonize the colon and cause gastrointestinal diseases, offering a potential solution for these types of infections.

## Figures and Tables

**Table 1:** Attributes and drawbacks of endolysins based on their name as antimicrobial.

Attributes		Disadvantages	
Specificity	Impossibility of dysbiosis [14,15,16]	Immunogenicity	Antibodies fail to neutralize endolysins in vivo due to strong CBD binding and rapid endolysin kinetics [13,17]
Activity	Peptidoglycan is rapidly degraded, lysing even inactive cells [18,19]	Half-life	Fast action and strong bond may be enough [7]
Possible resistance	Endopeptidases cleave at cross-bridges, and triple-action EAD endolysins reduce resistance [20,21]	Proinflammation	Continuous administration (proinflammatory cytokines such as TNF-α, IL-1ß, and IFN-γ) [22,23]
Resensitization	Antibiotic-resistant bacteria become sensitive to antibiotics by adding endolysin [24]	Intracellular bacteria not accessible	Endolysins kill intracellular bacteria, or fusion with transduction domains enables cell uptake [20,25]

**Table 3:** Abiotic factors that alter endolysin stability.

Endolisyn	Temperature	pH (Optimal)	Storage Details	References
KP27	Stable: 50–80 °C	2.6–10	10% loss of activity after 1 month storage T 4° C	[39]
PlyE146	–	Negligible activity above 7	–	[40]
PlyF307	–	6–8 (6)	–	[37]
KZ144	>50 °C gradually reduces its activity (60 °C no activity)	4.5–9 (6.2–6.5)	4 months at 4 °C in enzymatic buffer; the activity is maintained	[14]
SPN9CC	Active: 24–65 °C Optimal: 50–55 °C	6–10 (7.5–8.5)	–	[43]
AcLys	50% de perdida de actividad a 37 °C en 2 h	5–8 (6)	–	[41]
LysPA26	Active: 4–100 °C Optimal: 37–50 °C	2–10 (7–8)	–	[38]
Ply6A3	Active: 22–42 °C Optimal: 32 °C	5.5–8.5 (7.5)	–	[42]
LysAB2	Stable: 20–40 °C	4–8 (6)	–	[36]

**Table 4:** Pharmaceutical products with endolysins as active agent.

Company	Identifier	Bacterial Target	Endolisin(s)
Lysando	–	Bacterial infection of the wound	Medolysin^®^
Intron Biotechnology	NCT03089697	Staphylococcal infections	SAL200 (N-Rephasin^®^)
Micreos	NCT02840955	Staphylococcal infections	Gladskin (Staphefekt ^TM^ XDR.300)
Contrafect	NCT03163446	*S. aureus* bacteremia	CF-301
Gangagen	NCT01746654	*S. aureus* in nasal environments	P128

**Table 5:** Antimicrobial effectiveness of biocomposites.

Material	Inhibition Spectrum	Method	References
Functionalized polyurethane	*S. aureus* *E. coli* *Vancomycin-resistant Enterococcus*	Incubation broth (5 × 10^7^ CFU/mL) at 37 °C for 16 h	[56]
Functionalized polyurethane	*S. aureus* *S. epidermis* *P. aeruginosa* *E. coli*	Submerged polymer incubation of 10^6^ CFU/mL for 48 h at 37 °C	[57]
Functionalized silicone	*S. aureus* *E. coli* *P. aeruginosa*	10^8^ CFU/mL droplets on polymer for 3 h at 37 °C with humidity	[58]
Functionalized poly-lactic acid	*E. coli* *Bacillus subtilis*	Overnight cultures at 0.07 OD_600_, 24 h drop in contact with polymer	[59]
Amphiphilic polymers	*P. aeruginosa* *E. coli* *S. aureus*	Broth dilution method of polymer samples using MIC	[60]

**Table 6:** Physiological effects of short-chain fatty acids (SCFAs).

Short Chain Fatty Acid (SCFA)	Physiological Effect	Details	References
Acetic acid (AA)	Nutritional	Obtaining energy via the Krebs cycle	[67]
	Antinutritional	Promotes cholesterol biosynthesis due to high acetate content in serum	[67]
	Immunomodulator	Interacts with guanosine triphosphate (GTP) binding proteins on immune cells	[68,69]
	Anti-inflammatory	Decreases lipopolysaccharide (LPS)-stimulated tumor necrosis factor (TNFα) release	[68]
	Anticancer	Inhibits NF-κB reporter activity in human colon carcinoma	[68]
	Colonic function	Increases colonic blood flow	[70]
Propionic acid (PA)	Nutritional	Obtaining energy via gluconeogenesis	[67]
	Nutritional	Inhibition of cholesterol biosynthesis due to high serum propionate content	[67]
	Nutritional	Lowers blood glucose and alters lipid metabolism in healthy human subjects	[71]
	Antiinflammatory	Propionate derivatives inhibit cyclooxygenase activity, involved in the production of proinflammatory species	[72]
	Antiinflammatory	Inhibits LPS-stimulated TNF-α production in human neutrophils	[68]
	Satiety	Propionate dietary supplementation by fermentation of a dairy beverage with propionic acid bacteria increased satiety in humans	[73]
	Satiety	Increased production of leptin, a satiety hormone.	[74]
	Neurological	Propionate infusion results in altered brain phospholipid and acylcarnitine profiles	[75]
	Neurological	Induced neuroinflammation and oxidative stress in brain regions in rats with intraventricular infusion	[76]
	Colonic function	Concentration-dependent increase in the frequency of spontaneous contractions in longitudinal and circular smooth muscle of the colon	[77]
Butyric acid (BA)	Nutritional	Colonocytes use butyrate as their main source of energy	[78]
	Nutritional	Maintains normal rates of colonocyte growth and proliferation	[78]
	Anticancer	Inhibition of colorectal cancer cell (CRC) growth and induction of apoptosis is triggered by histone deacetylase (HDAC) inhibition	[79]
	Detoxifying	CRC cells treated in vitro with butyrate overexpressed enzymes involved in the defense against genotoxic and mutagenic agents, indicating a protective effect of butyrate also at the level of detoxifying enzymes	[80]
	Prevention of intestinal diseases (colitis)	Butyrate overproduction upregulates mucin gene expression, which protects mucosal surfaces and prevents increased cell permeability	[81]
	Anti-inflammatory	Reduction of the levels of proinflammatory mediators and stimulate the production of immunosuppressive cytokines	[82]
	Anti-inflammatory	Increased endogenous production of GLP-2 (glucagon-like peptide-2). This peptide significantly reduces the concentration of pro-inflammatory factors in the blood	[83]
	Immunomodulator	Decreases Interleukin-12 (IL-12) expression and increases Interleukin-10 (IL-10) production in human monocytes	[84]
	Anticancer	Modulation of genes associated with proliferation, differentiation, and apoptosis in colonic epithelial cells	[85]
	Antioxidant	In vitro modulation of oxidative and metabolic stress genes in human colon cells	[86]
	Satiety	Butyrate can increase the expression of peptides involved in the regulation of appetite	[87]
	Colonic function	Regulates enteric neurons and controls intestinal motility	[88]
	Insulin resistance	May reverse and prevent diet-induced insulin resistance	[89]

## Data Availability

The data presented in this study are available on request from the corresponding author.

## References

[1] Kongkham B., Prabakaran D., Puttaswamy H. (2020). Opportunities and challenges in managing antibiotic resistance in bacteria using plant secondary metabolites. Fitoterapia.

[2] Jindal A.K., Pandya K., Khan I.D. (2015). Antimicrobial resistance: A public health challenge. Med. J. Armed Forces India.

[3] Alós J.-I. (2015). Resistencia bacteriana a los antibióticos: Una crisis global. Enferm. Infecc. Microbiol. Clin..

[4] Whatmore A.M., Reed R.H. (1990). Determination of turgor pressure in Bacillus subtilis: A possible role for K+ in turgor regulation. J. Gen. Microbiol..

[5] Calero-Cáceres W., Ye M., Balcázar J.L. (2019). Bacteriophages as Environmental Reservoirs of Antibiotic Resistance. Trends Microbiol..

[6] Rahman M.U., Wang W., Sun Q., Shah J.A., Li C., Sun Y., Li Y., Zhang B., Chen W., Wang S. (2021). Endolysin, a Promising Solution against Antimicrobial Resistance. Antibiotics.

[7] Gerstmans H., Criel B., Briers Y. (2018). Synthetic biology of modular endolysins. Biotechnol. Adv..

[8] Camacho-González C.E., Cardona-Félix C.S., Zamora-Gasga V., Pérez-Larios A., Sánchez-Burgos J.A. (2022). Biofunctionalization of Endolysins with Oligosacharides: Formulation of Therapeutic Agents to Combat Multi-Resistant Bacteria and Potential Strategies for Their Application. Polysaccharides.

[9] SUIVE/DGE/SSA (2022). Veinte Principales Causas de Enfermedad Nacional en México. https://epidemiologia.salud.gob.mx/anuario/html/morbilidad_nacional.html.

[10] Gondil V.S., Harjai K., Chhibber S. (2020). Endolysins as emerging alternative therapeutic agents to counter drug-resistant infections. Int. J. Antimicrob. Agents.

[11] Young R. (1992). Bacteriophage lysis: Mechanism and regulation. Microbiol. Rev..

[12] Wu X.S., Wang N. (2001). Synthesis, characterization, biodegradation, and drug delivery application of biodegradable lactic/glycolic acid polymers. Part II: Biodegradation. J. Biomater. Sci. Polym. Ed..

[13] Loessner M.J., Kramer K., Ebel F., Scherer S. (2002). C-terminal domains of Listeria monocytogenes bacteriophage murein hydrolases determine specific recognition and high-affinity binding to bacterial cell wall carbohydrates. Mol. Microbiol..

[14] Briers Y., Volckaert G., Cornelissen A., Lagaert S., Michiels C.W., Hertveldt K., Lavigne R. (2007). Muralytic activity and modular structure of the endolysins of Pseudomonas aeruginosa bacteriophages φKZ and ELs. Mol. Microbiol..

[15] Schleifer K.H., Kandler O. (1972). Peptidoglycan types of bacterial cell walls and their taxonomic implications. Bacteriol. Rev..

[16] Nelson D.C., Schmelcher M., Rodriguez-Rubio L., Klumpp J., Pritchard D.G., Dong S., Donovan D.M. (2012). Endolysins as Antimicrobials. Adv. Virus Res..

[17] Jado I. (2003). Phage lytic enzymes as therapy for antibiotic-resistant Streptococcus pneumoniae infection in a murine sepsis model. J. Antimicrob. Chemother..

[18] Gutiérrez D., Ruas-Madiedo P., Martínez B., Rodríguez A., García P. (2014). Effective Removal of Staphylococcal Biofilms by the Endolysin LysH5. PLoS ONE.

[19] Briers Y., Walmagh M., Grymonprez B., Biebl M., Pirnay J.-P., Defraine V., Michiels J., Cenens W., Aertsen A., Miller S. (2014). Art-175 Is a Highly Efficient Antibacterial against Multidrug-Resistant Strains and Persisters of Pseudomonas aeruginosa. Antimicrob. Agents Chemother..

[20] Becker S.C., Roach D.R., Chauhan V.S., Shen Y., Foster-Frey J., Powell A.M., Bauchan G., Lease R.A., Mohammadi H., Harty W.J. (2016). Triple-acting Lytic Enzyme Treatment of Drug-Resistant and Intracellular Staphylococcus aureus. Sci. Rep..

[21] Fernández L., González S., Campelo A.B., Martínez B., Rodríguez A., García P. (2017). Downregulation of Autolysin-Encoding Genes by Phage-Derived Lytic Proteins Inhibits Biofilm Formation in Staphylococcus aureus. Antimicrob. Agents Chemother..

[22] Witzenrath M., Schmeck B., Doehn J.M., Tschernig T., Zahlten J., Loeffler J.M., Zemlin M., Müller H., Gutbier B., Schütte H. (2009). Systemic use of the endolysin Cpl-1 rescues mice with fatal pneumococcal pneumonia*. Crit. Care Med..

[23] Zhang L., Li D., Li X., Hu L., Cheng M., Xia F., Gong P., Wang B., Ge J., Zhang H. (2016). LysGH15 kills Staphylococcus aureus without being affected by the humoral immune response or inducing inflammation. Sci. Rep..

[24] Djurkovic S., Loeffler J.M., Fischetti V.A. (2005). Synergistic Killing of *Streptococcus pneumoniae* with the Bacteriophage Lytic Enzyme Cpl-1 and Penicillin or Gentamicin Depends on the Level of Penicillin Resistance. Antimicrob. Agents Chemother..

[25] Shen Y., Barros M., Vennemann T., Gallagher D.T., Yin Y., Linden S.B., Heselpoth R.D., Spencer D.J., Donovan D.M., Moult J. (2016). A bacteriophage endolysin that eliminates intracellular streptococci. Elife.

[26] Zermeño-Cervantes L.A., González-Acosta B., Martínez-Díaz S.F., Cardona-Félix C.S. (2020). Antibacterial proteins and peptides as potential treatment in aquaculture: Current status and perspectives on delivery. Rev. Aquac..

[27] Breijyeh Z., Jubeh B., Karaman R. (2020). Resistance of Gram-Negative Bacteria to Current Antibacterial Agents and Approaches to Resolve It. Molecules.

[28] World Health Organization (2024). WHO Bacterial Priority Pathogens List, 2024: Bacterial Pathogens of Public Health Importance to Guide Research, Development and Strategies to Prevent and Control Antimicrobial Resistance.

[29] Liu H., Hu Z., Li M., Yang Y., Lu S., Rao X. (2023). Therapeutic potential of bacteriophage endolysins for infections caused by Gram-positive bacteria. J. Biomed. Sci..

[30] Seal B.S. (2013). Characterization of bacteriophages virulent for Clostridium perfringens and identification of phage lytic enzymes as alternatives to antibiotics for potential control of the bacterium. Poult. Sci..

[31] Swift S., Seal B., Garrish J., Oakley B., Hiett K., Yeh H.-Y., Woolsey R., Schegg K., Line J., Donovan D. (2015). A Thermophilic Phage Endolysin Fusion to a Clostridium perfringens-Specific Cell Wall Binding Domain Creates an Anti-Clostridium Antimicrobial with Improved Thermostability. Viruses.

[32] Simmons M., Morales C.A., Oakley B.B., Seal B.S. (2012). Recombinant Expression of a Putative Amidase Cloned from the Genome of Listeria monocytogenes that Lyses the Bacterium and its Monolayer in Conjunction with a Protease. Probiotics Antimicrob. Proteins.

[33] Schmelcher M., Shen Y., Nelson D.C., Eugster M.R., Eichenseher F., Hanke D.C., Loessner M.J., Dong S., Pritchard D.G., Lee J.C. (2015). Evolutionarily distinct bacteriophage endolysins featuring conserved peptidoglycan cleavage sites protect mice from MRSA infection. J. Antimicrob. Chemother..

[34] Son B., Yun J., Lim J.-A., Shin H., Heu S., Ryu S. (2012). Characterization of LysB4, an endolysin from the Bacillus cereus-infecting bacteriophage B4. BMC Microbiol..

[35] Lv M., Wang S., Yan G., Sun C., Feng X., Gu J., Han W., Lei L. (2015). Genome sequencing and analysis of an Escherichia coli phage vB_EcoM-ep3 with a novel lysin, Lysep3. Virus Genes.

[36] Lai M.-J., Lin N.-T., Hu A., Soo P.-C., Chen L.-K., Chen L.-H., Chang K.-C. (2011). Antibacterial activity of Acinetobacter baumannii phage ϕAB2 endolysin (LysAB2) against both Gram-positive and Gram-negative bacteria. Appl. Microbiol. Biotechnol..

[37] Lood R., Winer B.Y., Pelzek A.J., Diez-Martinez R., Thandar M., Euler C.W., Schuch R., Fischetti V.A. (2015). Novel Phage Lysin Capable of Killing the Multidrug-Resistant Gram-Negative Bacterium Acinetobacter baumannii in a Mouse Bacteremia Model. Antimicrob. Agents Chemother..

[38] Guo M., Feng C., Ren J., Zhuang X., Zhang Y., Zhu Y., Dong K., He P., Guo X., Qin J. (2017). A Novel Antimicrobial Endolysin, LysPA26, against Pseudomonas aeruginosa. Front. Microbiol..

[39] Maciejewska B., Roszniowski B., Espaillat A., Kęsik-Szeloch A., Majkowska-Skrobek G., Kropinski A.M., Briers Y., Cava F., Lavigne R., Drulis-Kawa Z. (2017). Klebsiella phages representing a novel clade of viruses with an unknown DNA modification and biotechnologically interesting enzymes. Appl. Microbiol. Biotechnol..

[40] Larpin Y., Oechslin F., Moreillon P., Resch G., Entenza J.M., Mancini S. (2018). In vitro characterization of PlyE146, a novel phage lysin that targets Gram-negative bacteria. PLoS ONE.

[41] Sykilinda N., Nikolaeva A., Shneider M., Mishkin D., Patutin A., Popov V., Boyko K., Klyachko N., Miroshnikov K. (2018). Structure of an Acinetobacter Broad-Range Prophage Endolysin Reveals a C-Terminal α-Helix with the Proposed Role in Activity against Live Bacterial Cells. Viruses.

[42] Wu M., Hu K., Xie Y., Liu Y., Mu D., Guo H., Zhang Z., Zhang Y., Chang D., Shi Y. (2019). A Novel Phage PD-6A3, and Its Endolysin Ply6A3, With Extended Lytic Activity Against Acinetobacter baumannii. Front. Microbiol..

[43] Lim J.-A., Shin H., Heu S., Ryu S. (2014). Exogenous Lytic Activity of SPN9CC Endolysin Against Gram-Negative Bacteria. J. Microbiol. Biotechnol..

[44] Rodríguez-Rubio L., Gerstmans H., Thorpe S., Mesnage S., Lavigne R., Briers Y. (2016). DUF3380 Domain from a Salmonella Phage Endolysin Shows Potent *N* -Acetylmuramidase Activity. Appl. Environ. Microbiol..

[45] Yang Y., Le S., Shen W., Chen Q., Huang Y., Lu S., Tan Y., Li M., Hu F., Li Y. (2018). Antibacterial Activity of a Lytic Enzyme Encoded by Pseudomonas aeruginosa Double Stranded RNA Bacteriophage phiYY. Front. Microbiol..

[46] Legotsky S.A., Vlasova K.Y., Priyma A.D., Shneider M.M., Pugachev V.G., Totmenina O.D., Kabanov A.V., Miroshnikov K.A., Klyachko N.L. (2014). Peptidoglycan degrading activity of the broad-range Salmonella bacteriophage S-394 recombinant endolysin. Biochimie.

[47] Thummeepak R., Kitti T., Kunthalert D., Sitthisak S. (2016). Enhanced Antibacterial Activity of Acinetobacter baumannii Bacteriophage ØABP-01 Endolysin (LysABP-01) in Combination with Colistin. Front. Microbiol..

[48] Oliveira H., Vilas Boas D., Mesnage S., Kluskens L.D., Lavigne R., Sillankorva S., Secundo F., Azeredo J. (2016). Structural and Enzymatic Characterization of ABgp46, a Novel Phage Endolysin with Broad Anti-Gram-Negative Bacterial Activity. Front. Microbiol..

[49] Díez-Martínez R., de Paz H., Bustamante N., García E., Menéndez M., García P. (2013). Improving the Lethal Effect of Cpl-7, a Pneumococcal Phage Lysozyme with Broad Bactericidal Activity, by Inverting the Net Charge of Its Cell Wall-Binding Module. Antimicrob. Agents Chemother..

[50] Briers Y., Walmagh M., Van Puyenbroeck V., Cornelissen A., Cenens W., Aertsen A., Oliveira H., Azeredo J., Verween G., Pirnay J.-P. (2014). Engineered Endolysin-Based “Artilysins” To Combat Multidrug-Resistant Gram-Negative Pathogens. MBio.

[51] Abouhmad A., Mamo G., Dishisha T., Amin M.A., Hatti-Kaul R. (2016). T4 lysozyme fused with cellulose-binding module for antimicrobial cellulosic wound dressing materials. J. Appl. Microbiol..

[52] Bai J., Yang E., Chang P.-S., Ryu S. (2019). Preparation and characterization of endolysin-containing liposomes and evaluation of their antimicrobial activities against gram-negative bacteria. Enzyme Microb. Technol..

[53] Yadav M.K., Kumari I., Singh B., Sharma K.K., Tiwari S.K. (2022). Probiotics, prebiotics and synbiotics: Safe options for next-generation therapeutics. Appl. Microbiol. Biotechnol..

[54] Muhamad I.I., Lazim N.A.M., Selvakumaran S. (2019). Natural polysaccharide-based composites for drug delivery and biomedical applications. Natural Polysaccharides in Drug Delivery and Biomedical Applications.

[55] Liu M., Bauman L., Nogueira C.L., Aucoin M.G., Anderson W.A., Zhao B. (2022). Antimicrobial polymeric composites for high-touch surfaces in healthcare applications. Curr. Opin. Biomed. Eng..

[56] Liu Q., Zhang Y., Liu W., Wang L., Choi Y.W., Fulton M., Fuchs S., Shariati K., Qiao M., Bernat V. (2021). A Broad-Spectrum Antimicrobial and Antiviral Membrane Inactivates SARS-CoV-2 in Minutes. Adv. Funct. Mater..

[57] Wo Y., Xu L.-C., Li Z., Matzger A.J., Meyerhoff M.E., Siedlecki C.A. (2017). Antimicrobial nitric oxide releasing surfaces based on S-nitroso-N-acetylpenicillamine impregnated polymers combined with submicron-textured surface topography. Biomater. Sci..

[58] Li Z., Liu H., Xu X., Ma L., Shang S., Song Z. (2020). Surface modification of silicone elastomer with rosin acid-based quaternary ammonium salt for antimicrobial and biocompatible properties. Mater. Des..

[59] Ojogbo E., Ward V., Mekonnen T.H. (2020). Functionalized starch microparticles for contact-active antimicrobial polymer surfaces. Carbohydr. Polym..

[60] Pham P., Oliver S., Wong E.H.H., Boyer C. (2021). Effect of hydrophilic groups on the bioactivity of antimicrobial polymers. Polym. Chem..

[61] Faria-Tischer P.C.S., Ribeiro-Viana R.M., Tischer C.A. (2019). Bio-based nanocomposites. Materials for Biomedical Engineering.

[62] Chen D., Bai R., Yong H., Zong S., Jin C., Liu J. (2022). Improving the digestive stability and prebiotic effect of carboxymethyl chitosan by grafting with gallic acid: In vitro gastrointestinal digestion and colonic fermentation evaluation. Int. J. Biol. Macromol..

[63] Macfarlane G.T., Steed H., Macfarlane S. (2007). Bacterial metabolism and health-related effects of galacto-oligosaccharides and other prebiotics. J. Appl. Microbiol..

[64] Macfarlane G.T., Macfarlane S. (2012). Bacteria, Colonic Fermentation, and Gastrointestinal Health. J. AOAC Int..

[65] Fernández J., Redondo-Blanco S., Gutiérrez-del-Río I., Miguélez E.M., Villar C.J., Lombó F. (2016). Colon microbiota fermentation of dietary prebiotics towards short-chain fatty acids and their roles as anti-inflammatory and antitumour agents: A review. J. Funct. Foods.

[66] LaBouyer M., Holtrop G., Horgan G., Gratz S.W., Belenguer A., Smith N., Walker A.W., Duncan S.H., Johnstone A.M., Louis P. (2022). Higher total faecal short-chain fatty acid concentrations correlate with increasing proportions of butyrate and decreasing proportions of branched-chain fatty acids across multiple human studies. Gut Microbiome.

[67] Wong J.M.W., de Souza R., Kendall C.W.C., Emam A., Jenkins D.J.A. (2006). Colonic Health: Fermentation and Short Chain Fatty Acids. J. Clin. Gastroenterol..

[68] Tedelind S., Westberg F., Kjerrulf M., Vidal A. (2007). Anti-inflammatory properties of the short-chain fatty acids acetate and propionate: A study with relevance to inflammatory bowel disease. World J. Gastroenterol..

[69] Brown A.J., Goldsworthy S.M., Barnes A.A., Eilert M.M., Tcheang L., Daniels D., Muir A.I., Wigglesworth M.J., Kinghorn I., Fraser N.J. (2003). The Orphan G Protein-coupled Receptors GPR41 and GPR43 Are Activated by Propionate and Other Short Chain Carboxylic Acids. J. Biol. Chem..

[70] Scheppach W. (1994). Effects of short chain fatty acids on gut morphology and function. Gut.

[71] Todesco T., Rao A.V., Bosello O., Jenkins D.J. (1991). Propionate lowers blood glucose and alters lipid metabolism in healthy subjects. Am. J. Clin. Nutr..

[72] Dannhardt G., Lehr M. (1993). Nonsteriodal Antiinflammatory Agents, XVII: Inhibition of Bovine Cyclooxygenase and 5-Lipoxygenase byN-Alkyldiphenyl-pyrrolyl Acetic and Propionic Acid Derivatives. Arch. Pharm..

[73] Ruijschop R.M.A.J., Boelrijk A.E.M., te Giffel M.C. (2008). Satiety effects of a dairy beverage fermented with propionic acid bacteria. Int. Dairy J..

[74] Xiong Y., Miyamoto N., Shibata K., Valasek M.A., Motoike T., Kedzierski R.M., Yanagisawa M. (2004). Short-chain fatty acids stimulate leptin production in adipocytes through the G protein-coupled receptor GPR41. Proc. Natl. Acad. Sci. USA.

[75] Thomas R.H., Foley K.A., Mepham J.R., Tichenoff L.J., Possmayer F., MacFabe D.F. (2010). Altered brain phospholipid and acylcarnitine profiles in propionic acid infused rodents: Further development of a potential model of autism spectrum disorders. J. Neurochem..

[76] MacFabe D.F., Rodriguez- K., Hoffman J.E., Franklin A.E., Mohammad-Asef Y., Taylor A.R., Boon F., Cain D.P., Kavaliers M., Possmayer F. (2008). A Novel Rodent Model of Autism: Intraventricular Infusions of Propionic Acid Increase Locomotor Activity and Induce Neuroinflammation and Oxidative Stress in Discrete Regions of Adult Rat Brain. Am. J. Biochem. Biotechnol..

[77] Ono S., Karaki S., Kuwahara A. (2004). Short-Chain Fatty Acids Decrease the Frequency of Spontaneous Contractions of Longitudinal Muscle via Enteric Nerves in Rat Distal Colon. Jpn. J. Physiol..

[78] Cruz-Bravo R.K., Guevara-González R.G., Ramos-Gómez M., Oomah B.D., Wiersma P., Campos-Vega R., Loarca-Piña G. (2014). The fermented non-digestible fraction of common bean (*Phaseolus vulgaris* L.) triggers cell cycle arrest and apoptosis in human colon adenocarcinoma cells. Genes Nutr..

[79] Heerdt B., Houston M., Augenlicht L. (1997). Short-chain fatty acid-initiated cell cycle arrest and apoptosis of colonic epithelial cells is linked to mitochondrial function. Cell Growth Differ..

[80] Stein K., Borowicki A., Scharlau D., Schettler A., Scheu K., Obst U., Glei M. (2012). Effects of synbiotic fermentation products on primary chemoprevention in human colon cells. J. Nutr. Biochem..

[81] Hudcovic T., Kolinska J., Klepetar J., Stepankova R., Rezanka T., Srutkova D., Schwarzer M., Erban V., Du Z., Wells J.M. (2012). Protective effect of *Clostridium tyrobutyricum* in acute dextran sodium sulphate-induced colitis: Differential regulation of tumour necrosis factor-α and interleukin-18 in BALB/c and severe combined immunodeficiency mice. Clin. Exp. Immunol..

[82] Wasilewski A., Zielińska M., Storr M., Fichna J. (2015). Beneficial Effects of Probiotics, Prebiotics, Synbiotics, and Psychobiotics in Inflammatory Bowel Disease. Inflamm. Bowel Dis..

[83] Villanueva-Millán M.J., Pérez-Matute P., Oteo J.A. (2015). Gut microbiota: A key player in health and disease. A review focused on obesity. J. Physiol. Biochem..

[84] Säemann M.D., Böhmig G.A., Österreicher C.H., Burtscher H., Parolini O., Diakos C., Stöckl J., Hörl W.H., Zlabinger G.J. (2000). Anti-inflammatory effects of sodium butyrate on human monocytes: Potent inhibition of IL-12 and up-regulation of IL-10 production. FASEB J..

[85] Daly K., Shirazi-Beechey S.P. (2006). Microarray Analysis of Butyrate Regulated Genes in Colonic Epithelial Cells. DNA Cell Biol..

[86] Sauer J., Richter K.K., Pool-Zobel B.L. (2007). Physiological concentrations of butyrate favorably modulate genes of oxidative and metabolic stress in primary human colon cells. J. Nutr. Biochem..

[87] Zhou J., Hegsted M., McCutcheon K.L., Keenan M.J., Xi X., Raggio A.M., Martin R.J. (2006). Peptide YY and Proglucagon mRNA Expression Patterns and Regulation in the Gut*. Obesity.

[88] Soret R., Chevalier J., De Coppet P., Poupeau G., Derkinderen P., Segain J.P., Neunlist M. (2010). Short-Chain Fatty Acids Regulate the Enteric Neurons and Control Gastrointestinal Motility in Rats. Gastroenterology.

[89] Gao Z., Yin J., Zhang J., Ward R.E., Martin R.J., Lefevre M., Cefalu W.T., Ye J. (2009). Butyrate Improves Insulin Sensitivity and Increases Energy Expenditure in Mice. Diabetes.

[90] Souza C.J.F., Comunian T.A., Kasemodel M.G.C., Favaro-Trindade C.S. (2019). Microencapsulation of lactase by W/O/W emulsion followed by complex coacervation: Effects of enzyme source, addition of potassium and core to shell ratio on encapsulation efficiency, stability and kinetics of release. Food Res. Int..

[91] Boi S., Dellacasa E., Bianchini P., Petrini P., Pastorino L., Monticelli O. (2019). Encapsulated functionalized stereocomplex PLA particles: An effective system to support mucolytic enzymes. Colloids Surfaces B Biointerfaces.

[92] Jiang F., Yang L., Wang S., Ying X., Ling J., Ouyang X. (2021). Fabrication and characterization of zein-alginate oligosaccharide complex nanoparticles as delivery vehicles of curcumin. J. Mol. Liq..

[93] Mrudulakumari Vasudevan U., Lee O.K., Lee E.Y. (2021). Alginate derived functional oligosaccharides: Recent developments, barriers, and future outlooks. Carbohydr. Polym..

[94] Liu M., Liu L., Zhang H., Yi B., Everaert N. (2021). Alginate oligosaccharides preparation, biological activities and their application in livestock and poultry. J. Integr. Agric..

[95] Hu X., Jiang X., Gong J., Hwang H., Liu Y., Guan H. (2005). Antibacterial activity of lyase-depolymerized products of alginate. J. Appl. Phycol..

[96] CDC Preliminary Incidence and Trends of Infections with Pathogens Transmitted Commonly Through Food. https://www.cdc.gov/foodnet/reports/prelim-data-intro-2018.html.

[97] Wang Y., Han F., Hu B., Li J., Yu W. (2006). In vivo prebiotic properties of alginate oligosaccharides prepared through enzymatic hydrolysis of alginate. Nutr. Res..

[98] Wan J., Zhang J., Chen D., Yu B., Huang Z., Mao X., Zheng P., Yu J., He J. (2020). Alterations in intestinal microbiota by alginate oligosaccharide improve intestinal barrier integrity in weaned pigs. J. Funct. Foods.

[99] Funami T., Fang Y., Noda S., Ishihara S., Nakauma M., Draget K.I., Nishinari K., Phillips G.O. (2009). Rheological properties of sodium alginate in an aqueous system during gelation in relation to supermolecular structures and Ca^2+^ binding. Food Hydrocoll..

[100] Rajwade J.M., Paknikar K.M., Kumbhar J.V. (2015). Applications of bacterial cellulose and its composites in biomedicine. Appl. Microbiol. Biotechnol..

[101] Lu S., Mikkelsen D., Flanagan B.M., Williams B.A., Gidley M.J. (2021). Interaction of cellulose and xyloglucan influences in vitro fermentation outcomes. Carbohydr. Polym..

[102] Stefanowski J., Weiss D. (2003). Carrot2 and Language Properties in Web Search Results Clustering. Advances in Web Intelligence.

[103] Melini F., Melini V., Luziatelli F., Ficca A.G., Ruzzi M. (2019). Health-Promoting Components in Fermented Foods: An Up-to-Date Systematic Review. Nutrients.

